# Digest: Great tits and their ticks: Life‐history traits affect tick fitness

**DOI:** 10.1111/evo.14543

**Published:** 2022-07-05

**Authors:** Kevin D. Matson, Willem F. de Boer, Jente Ottenburghs

**Affiliations:** ^1^ Wildlife Ecology and Conservation Wageningen University Wageningen 6700 AA The Netherlands; ^2^ Forest Ecology and Forest Management Wageningen University Wageningen 6700 AA The Netherlands

## Abstract

Ectoparasites such as ticks face many challenges to reproduce. They must maximize the size of their blood meal while avoiding being removed by their host. In a new study, Fracasso and colleagues followed the fate of individual ticks to determine which life‐history traits impact tick fitness. Their findings reveal a complex interplay between numerous parameters, including feeding time and engorgement weight. The situation is likely even more complicated when considering vector‐borne pathogens.

The emergence of infectious diseases continues to puzzle scientists. With vector‐borne diseases on the rise, studies on the ecology of ticks and mosquitoes are increasingly important to understand the factors and trade‐offs influencing the transmission of vector‐borne pathogens. For example, both weather and climate factors influence tick survival and reproduction (Randolph 2004; Boehnke et al. 2017). Moreover, ticks face the challenge of finding suitable hosts while having very limited movement capabilities (Randolph 1998). Complex trade‐offs govern vector biology: for example, the balance between maximizing the size of a blood meal and minimizing the possibility of being groomed off by a host. Documenting the fate of individual ticks—and the pathogens that they might carry—is an especially daunting challenge.

Recently, Fracasso et al. (2022a) reported on their mammoth efforts to breed *Ixodes arboricola* ticks and raise them over multiple life stages to investigate how certain tick life‐history traits influence the parasite's fitness. Tick life history was characterized using parameters such as feeding time and engorgement weight, and fitness was characterized using parameters related to tick success on and off the host (e.g., survival and feeding success). Using animal models, the authors also investigated the heritability and evolvability of these traits. These analyses resulted in an intricate and complex cause‐and‐effect diagram that highlights their findings (fig. 2 in Fracasso et al. 2022a). By feeding slowly, a tick increases its time on a host (and hence its chance of detection and removal) but surprisingly also reduces its engorgement weight. A lower engorgement weight has negative effects on the number of eggs produced. Hence, a large, quick blood meal seems to be the best strategy. Some of this phenotypic variation also has a genetic component, potentially allowing for long‐term adaptation to specific hosts.

The hide‐and‐seek dynamics playing out between ectoparasites and their hosts are complex. Fracasso et al. (2022a) have offered many new insights into the tick side of this study system, including measuring the effect that host individual characteristics have on the parasite (Fracasso et al. 2022b). These dynamics become even more complex if a vector‐borne pathogen is included in the system. These pathogens are, after all, the real culprits that make animals (including people) ill. For example, Belperron and Bockenstedt (2001) showed that innate immunological defenses (so‐called “natural antibodies”) of rodent hosts can actually reduce the fitness of the spirochete *Borrelia burgdorferi* when these antibodies and the pathogen come into contact *inside* feeding ticks, in this case *Ixodes scapularis*. Moreover, vector‐borne pathogens are able to increase or decrease the life span and questing behavior of their vectors (Hermann and Gern 2015; Benelli 2020). Furthermore, in response to tick bites, some (but apparently not all) mammals can produce specific antitick antibodies, which limit tick attachment time, engorgement, and molting success (Dizij and Kurtenbach 1995), some of the same aspects of tick biology studied by Fracasso et al. (2022a). One final wrinkle in this tripartite ecological interaction and co‐evolutionary arms race is that a mechanism used by ticks to defend themselves against the antibodies produced by their hosts (i.e., immunoglobulin binding proteins) may also offer protection to the *Borrelia* spirochetes within the ticks (Belperron and Bockenstedt 2001).

Taken together, these studies raise many questions about life‐history evolution and fitness in interacting pathogens, vectors, and hosts and, ultimately, about how such complex evolutionary dynamics lead to a costly toll on human health.

Associate Editor: Ms. Kati Moore

Handling Editor: Prof. Tracey Chapman

### SUBMIT A DIGEST

Digests are short (∼500 words), news articles about selected original research included in the journal, written by students or postdocs. These digests are published online and linked to their corresponding original research articles. For instructions on Digests preparation and submission, please visit the following link: https://sites.duke.edu/evodigests/.
